# miR-30e controls DNA damage-induced stress responses by modulating expression of the CDK inhibitor p21^WAF1/CIP1^ and caspase-3

**DOI:** 10.18632/oncotarget.7432

**Published:** 2016-02-17

**Authors:** Dennis Sohn, Dominik Peters, Roland P. Piekorz, Wilfried Budach, Reiner U. Jänicke

**Affiliations:** ^1^ Laboratory of Molecular Radiooncology, Clinic and Policlinic for Radiation Therapy and Radiooncology, Medical Faculty of the Heinrich-Heine-University, Düsseldorf, Germany; ^2^ Institute for Biochemistry and Molecular Biology II, Medical Faculty of the Heinrich-Heine-University, Düsseldorf, Germany

**Keywords:** apoptosis, senescence, microRNA-30e, p21, caspase-3

## Abstract

MicroRNAs (miRNAs), a class of small non-coding RNAs that usually cause gene silencing by translational repression or degradation of mRNAs, are implicated in DNA damage-induced stress responses. To identify senescence-associated miRNAs, we performed microarray analyses using wild-type and p53-deficient HCT116 colon carcinoma cells that following gamma-irradiation (γIR) are driven into senescence and apoptosis, respectively. Several miRNAs including miR-30e were found upregulated in a p53-dependent manner specifically in senescent cells, but not in apoptotic cells. Overexpression of miR-30e in HCT116 cells not only inhibited γIR-, etoposide- or miR-34a-induced caspase-3-like DEVDase activities and cell death, but greatly accelerated and augmented their senescent phenotype. Consistently, procaspase-3 protein, but not mRNA decreased in the presence of miR-30e, whereas expression of the cyclin-dependent kinase inhibitor p21 increased both at the mRNA and protein level. Performing luciferase reporter gene assays, we identified the 3′-UTR of the caspase-3 mRNA as a direct miR-30e target. In contrast, although miR-30e was unable to bind to the p21 mRNA, it increased expression of a luciferase construct containing the p21 promoter, suggesting that the miR-30e-mediated upregulation of p21 occurs indirectly at the transcriptional level. Interestingly, despite suppressing procaspase-3 expression, miR-30e was unable to protect RKO colon carcinoma cells from DNA damage-induced death or to induce senescence, as miR-30e completely fails to upregulate p21 in these cells. These data suggest that miR-30e functions in a cell type-dependent manner as an important molecular switch for DNA damage-induced stress responses and may thus represent a target of therapeutic value.

## INTRODUCTION

Cellular responses toward DNA-damaging agents such as chemotherapeutica and ionizing irradiation (gIR) are diverse and include induction of cell cycle checkpoints, DNA repair, programmed cell death and cellular senescence [1]. Central to these processes is the p53 protein that besides these tumor suppressor functions possesses also powerful oncogenic capabilities [2, 3]. Thus, mechanisms must exist which precisely govern p53-dependent decision processes between cellular survival and death. Although not yet fully elucidated, these include for instance co-factors that selectively modulate p53-dependent transcription, different promoter strengths of pro- versus anti-apoptotic target genes, differentially expressed p53 isoforms, as well as numerous post-translational modifications that together dictate the outcome of a p53 activation in a barcode-like manner [4–6].

One of the most prominent target genes transcriptionally induced by p53 is the *CDKN1A* gene encoding the cyclin-dependent kinase (CDK) inhibitor p21 that similar to p53 exhibits both pro- and anti-tumorigenic functions. This is because p21 blocks not only cell cycle progression resulting in either a temporary or permanent cell cycle arrest called senescence [7], but also inhibits apoptosis by several means [8, 9]. Thus, similar to p53, expression of p21 must be tightly controlled in order to avoid its untimely activation. This is achieved by diverse mechanisms acting at the transcriptional, translational, and post-translational level [10].

Although p53 is clearly a transcriptional activator, numerous reports indicate that it also represses certain genes and that such a repression is important for a proper function of this tumor suppressor [11, 12]. In addition, transcription-independent activities of p53 such as direct binding to anti-apoptotic Bcl-2 proteins at the mitochondria were reported [13], although particularly this model has been seriously challenged [14, 15]. The discovery of a novel class of small RNAs, so-called microRNAs (miRNAs), offers a plausible explanation for both these p53 ambiguities, as they not only repress target genes through the RNA interference pathway, but also because p53 controls expression of several miRNAs in a transcription-dependent and -independent manner [16].

MicroRNAs are short 20 – 22 nucleotide non-coding RNA molecules that regulate gene expression post-transcriptionally by targeting the 3′-UTRs of mRNAs [17]. A so-called “seed” region comprising bases 2 to 7 of the mature miRNA targets complementary mRNA sequences that thereby become either degraded or translationally repressed [18]. Comprising more than 1 000 members, miRNAs constitute one of the biggest gene family in the human genome. As individual miRNAs are capable of targeting hundreds of mRNAs [19], and because one miRNA can converge with others on a single target transcript [20], it is evident that these molecules play important roles in many biological processes including tumor development and progression. Indeed, multiple lines of genetic evidence indicate that miRNAs play key roles in mediating genotoxic stress responses [21]. Together with several functional studies, inappropriate miRNA expression profiles that have been found on a regular basis in a variety of tumor types, confirmed their classification into tumor suppressor miRNAs and oncogenic miRNAs [22, 23]. Prominent examples are the p53-regulated miR-34 family and the c-Myc-controlled miR-17-92 cluster that exert their tumor suppressive and oncogenic functions *via* direct translational repression of pro- (e.g. CDK4/6, cyclin D1, cyclin E2, c-Myc) and anti-proliferative (e.g. p21, p57) proteins, respectively [16, 24]. Thereby, miRNAs function not only downstream of p53 and c-Myc, both transcription factors are themselves under the control of miRNAs adding an additional layer of complexity to the multitude of signaling pathways controlled by them.

Comparing miRNA expression profiles of apoptotic and senescent HCT116 colon carcinoma cells, we show here that miR-30e is specifically upregulated in senescent cells controlling γIR-induced p53-dependent stress responses. By targeting pro- and anti-apoptotic proteins, miR-30e overrides apoptotic programming and redirects genotoxic signals specifically towards the induction of senescence. Thus, miR-30e might be an interesting therapeutic target particularly in tumors in which its upregulation contributes to therapy resistance.

## RESULTS

### Upregulation of miR30e in γIR-induced senescence

**Figure 1:**
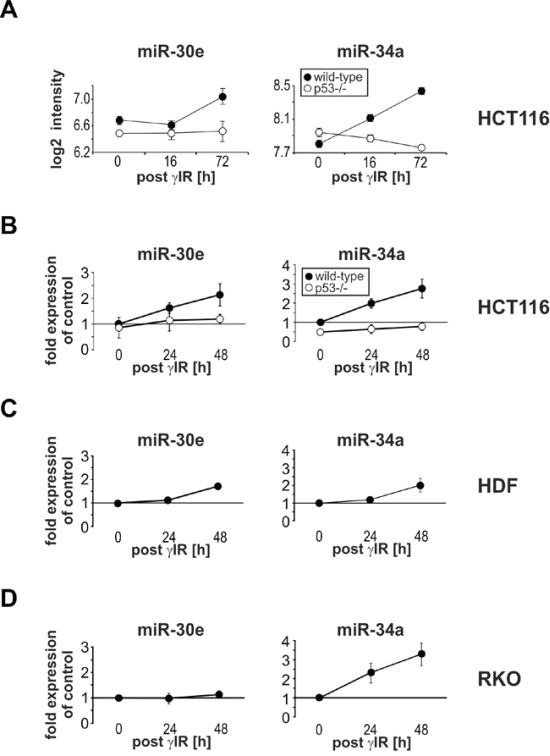
γIR induces miR-30e expression only in senescent HCT116 wild-type cells and HDF, but not in apoptotic p53-deficient HCT116 and RKO cells **A.** Microarray analyses for miR-34a and miR-30e expression in irradiated wild-type and p53-deficient HCT116 cells. **B-D.** Determination of miR-34a and miR-30e expression by real-time PCR analyses. Values shown in A to D are the mean of at least three independent experiments +/− S.D. Please note that statistical evaluations for the microarray and real-time PCR analyses using HCT116 cells can be found in Suppl. Figure 1.

In order to identify novel senescence-associated miRNAs, we performed a differential miRNA expression profiling via microarray analyses using gamma-irradiated (γIR) wild-type and p53-deficient HCT116 colon carcinoma cells [25]. We have chosen this particular cellular system because their fate upon γIR exposure critically depends on the presence or absence of p53 that is known to control a large network of stress-related miRNAs [16]. Whereas irradiated wild-type HCT116 cells are driven mainly into premature senescence due to the p53-dependent induction of p21, irradiated p53−/− HCT116 cells succumb to apoptosis as they are unable to upregulate this anti-apoptotic CDK inhibitor [9, 26]. Performing this screen, we identified several miRNAs including miR-30e that was upregulated by approximately 1.5-fold specifically in senescent wild-type cells when compared to apoptotic p53-deficient cells (Figure 1A). Besides miR-30e, only miR-30c, but none of the other members of the miR-30 family (miR-30a, -30b, -30d) was found upregulated by γIR again only in senescent wild-type cells (Suppl. Figure 1). We also observed a γIR-induced p53-dependent upregulation of miR-193b in HCT116 wild-type cells, whereas miR-7 and miR-484 levels decreased (Suppl. Figure 1). Finally, and as expected, expression of the p53-regulated miR-34a was also greatly enhanced in a p53-dependent manner demonstrating the specificity of our microarray analyses (Figure 1A; Suppl. Figure 1). Our further studies focused on the elucidation of miR-30e function in DNA damage-induced stress responses, as this miRNA belongs to a family known to be involved in cellular senescence [27], and whose members are postulated to mainly target mRNAs of proteins involved in apoptosis, senescence, and cell cycle control [28].

**Figure 2:**
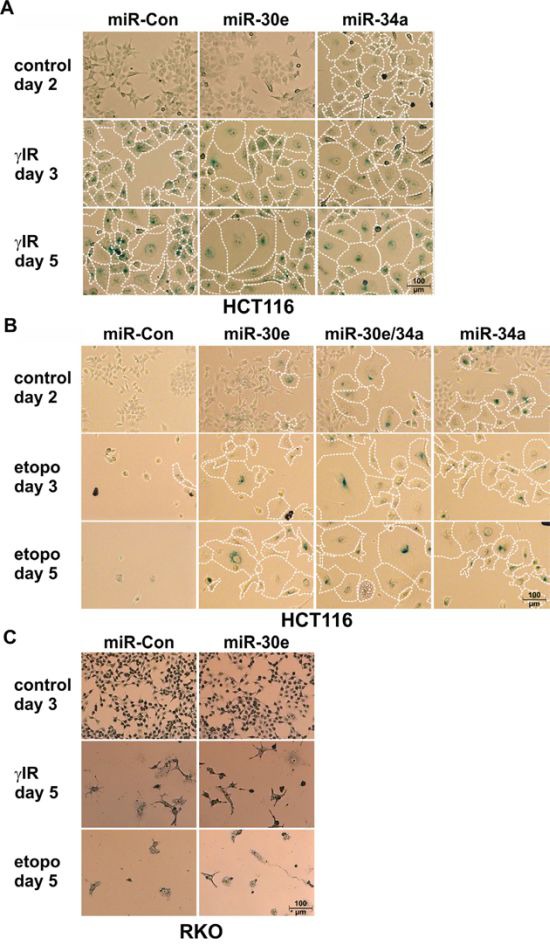
Effect of miR-30e on DNA damage-induced senescence **A-C.** MiR-30e accelerates and augments γIR-induced senescence of HCT116 wild-type cells. (A) and converts the etoposide-induced death program in these cells into senescence (B), but fails to function in a similar manner in RKO cells (C). Cells were transfected with the indicated miRNAs and were either left untreated (control) or exposed to γIR (A, C) or etoposide (B, C) and analyzed for SA-β-Gal staining at the indicated times. To better illustrate size increases, senescent SA-β-Gal-stained cells were encircled with dotted lines. Representative pictures out of at least three independent experiments are shown. All pictures were taken at a 10x magnification (100 μm scale bar included once in every panel). Please note that a quantification of the cell size increase of the treated HCT116 cells (A, B) is shown in Suppl. Figure 3.

**Figure 3:**
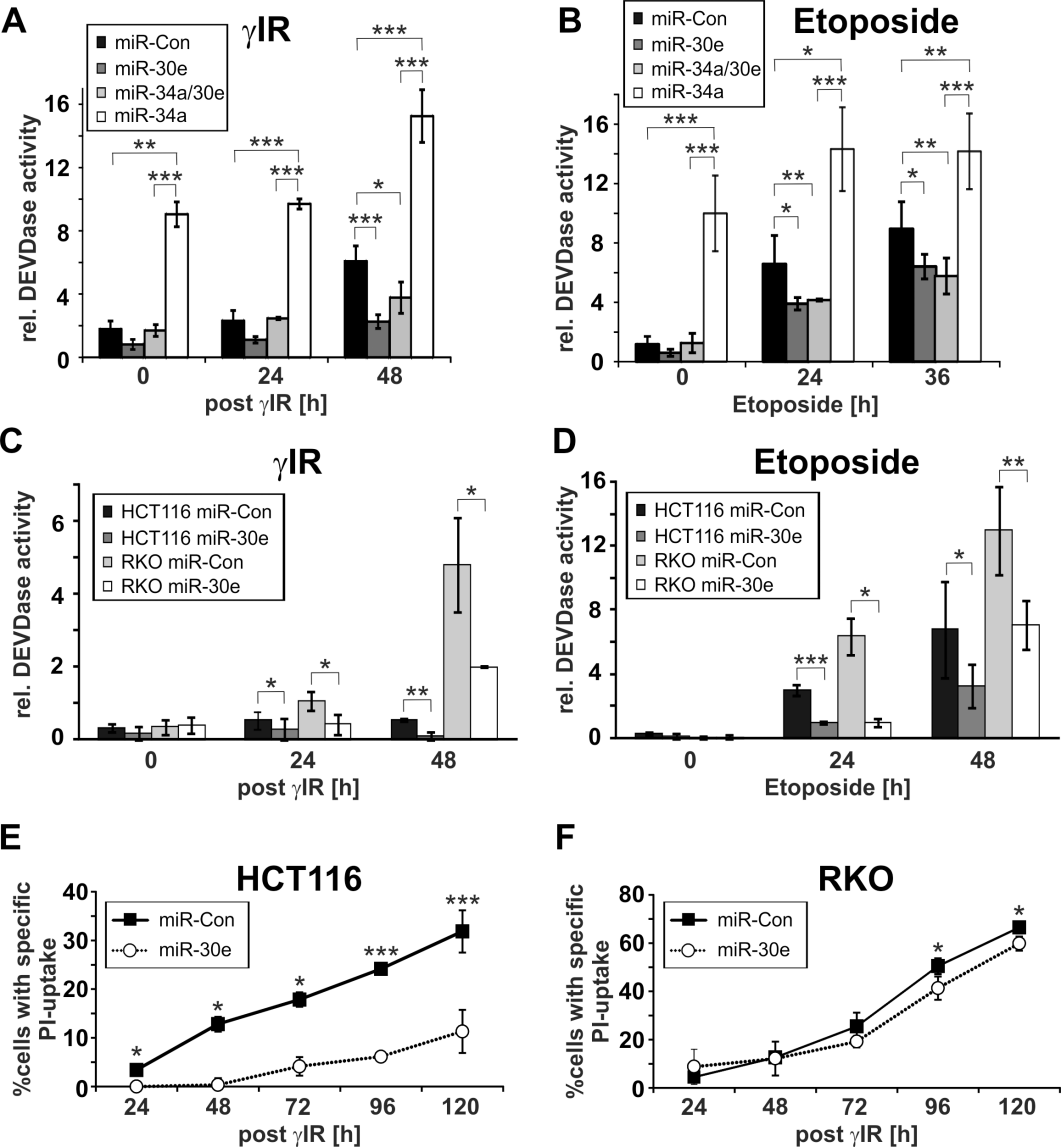
Effect of miR-30e on DNA damage-induced DEVDase activities and cell death **A-D.** MiR-30e inhibits DNA damage-induced DEVDase activities in HCT116 wild-type and RKO cells. DEVDase activities were determined in cells transfected with the indicated miRNAs at the indicated time points following exposure to γIR (A, C) or etoposide (B, D). **E, F.** MiR-30e inhibits γIR-induced cell death in HCT116 wild-type cells, but not in RKO cells. Death (PI uptake) of irradiated HCT116 wild-type (E) and RKO cells (F) transfected with miR-30e or a control miRNA (miR-Con) was determined by flow cytometric analyses at the indicated time points. Values shown in A to F are the mean of three to five independent experiments +/− S.D.

Real-time PCR analyses confirmed our microarray results demonstrating that miR-30e and miR-34a were specifically upregulated by γIR in a time- and dose-dependent manner only in wild-type but not in p53-deficient HCT116 cells, suggesting their p53-dependent expression (Figure 1B; Suppl. Figures 1, 2A, 2B). Both miRNAs were also found upregulated in gamma-irradiated senescent human dermal fibroblasts (HDF) (Figure 1C), whereas RKO colon carcinoma cells, despite their p53-proficiency, responded only with an increase of miR-34a (Figure 1D). Thus, for unknown reasons it appears that although p53 is required for γIR-induced miR-30e expression in HCT116 cells, it is not sufficient to induce expression of this miRNA in RKO cells. Interestingly, in contrast to wild-type HCT116 cells and HDF that both become senescent upon γIR exposure (Figure 2A and data not shown), irradiated RKO cells (like irradiated p53-deficient HCT116 cells [9, 26]) succumb to apoptosis as evidenced by increased caspase-3-like DEVDase activities (Figure 3C), elevated numbers of propidium iodide (PI)-stained dead cells (Figure 3F), and by the absence of senescence-associated β-galactidose (SA-β-Gal)-stained cells five days post γIR (Figure 2C). Thus, expression of miR-30e closely correlates with the induction of p53-dependent senescence.

### MiR-30e regulates DNA damage-induced stress responses in HCT116 cells

In contrast to miR-34a that is known to induce both apoptosis and senescence (Figures 2A, 2B, 3A, 3B; Suppl. Figure 3) [29], overexpression of miR-30e in either wild-type HCT116 or RKO cells had no effect on cell morphology and caspase-3-like activities in the absence of DNA damage (Figures 2, 3A-D; Suppl. Figure 3). In contrast, γIR exposure of miR-30e-transfected cells revealed remarkable differences. MiR-30e not only prevented γIR-induced background DEVDase activities in HCT116 wild-type cells, it almost completely inhibited caspase-3-like activities induced in these cells by miR-34a mimics alone or in combination with γIR (Figure 3A). Consistently, irradiation-induced cell death as measured by the uptake of PI was also significantly impaired in HCT116 cells overexpressing miR-30e mimics (Figure 3E). In addition, and most importantly, miR-30e greatly accelerated and augmented the senescent phenotype of irradiated HCT116 cells as evidenced by a vast increase in size and by an extremely flattened morphology of these cells compared to irradiated cells that were transfected with a control miRNA (Figure 2A; Suppl. Figure 3A). This phenomenon was comparable to that observed in irradiated miR-34a-transfected cells (Figure 2A; Suppl. Figure 3A), and was also evident in an irradiation dose-dependent manner when miR-30e-overexpressing HCT116 cells were exposed to γIR doses lower than 20 Gy such as 2 and 6 Gy (Suppl. Figure 2C).

Strikingly, compared with HCT116 cells transfected with a control miRNA, miR-30e overexpression not only inhibited the cell death mode normally executed by etoposide in these cells (Figures 3B; 3D), but in addition, converted this death signal into senescence which is most evident after five days of treatment (Figure 2B; Suppl. Figure 3B). MiR-30e prevented also miR-34a-induced DEVDase activities in both the absence and presence of etoposide (Figure 3B). Consistently, three days following exposure to etoposide, HCT116 cells co-transfected with miR-30e and miR-34a displayed an accelerated senescent phenotype when compared to etoposide-treated cells transfected with either miR alone (Figure 2B; Suppl. Figure 3B). Together, these data suggest that miR-30e represents a critical determinant in DNA damage-induced stress responses.

A different picture emerged when RKO cells, in which γIR fails to upregulate miR-30e (Figure 1D), were exposed to DNA-damaging agents. Although miR-30e mimics also inhibited γIR- or etoposide-induced activation of caspase-3-like proteases in these cells (Figures 3C, 3D), they were unable to prevent DNA damage-induced cell death (Figures 2C, 3F). Furthermore, and again unlike to the scenario observed in HCT116 cells, in RKO cells miR-30e completely failed to convert the death signals instigated by γIR or etoposide into the induction of senescence (Figure 2C). Thus, regardless of whether or not expression of miR-30e is inducible in RKO cells (Figure 1D), the observation that miR-30e converts the DNA damage-induced cell death programm into senescence only in HCT116 cells, but not in RKO cells, strongly suggests different miR-30e target usages in these cell lines.

### MiR-30e differentially targets p21 and caspase-3 expression in HCT116 and RKO cells

**Figure 4:**
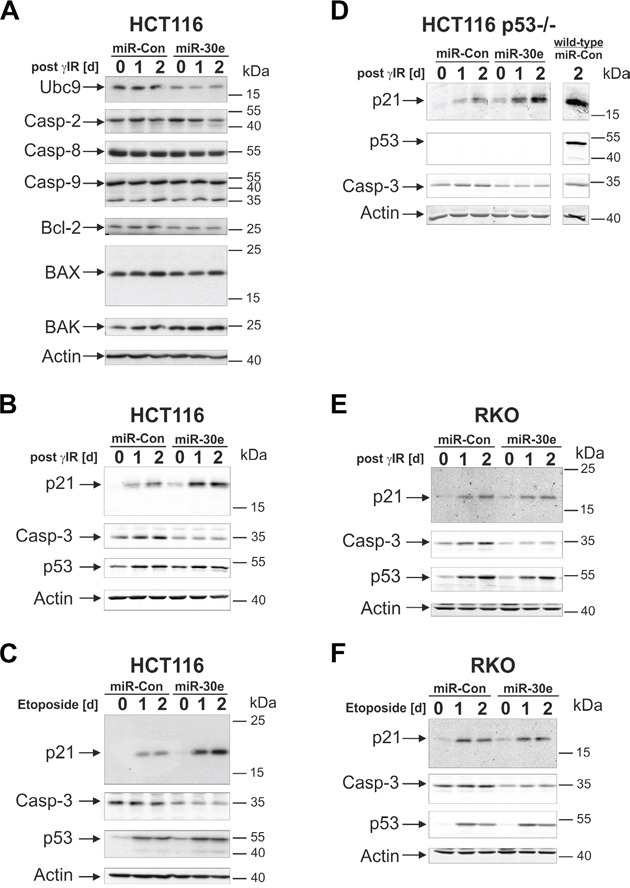
MiR-30e suppresses procaspase-3 expression in HCT116 and RKO cells, but upregulates p21 only in HCT116 cells **A-F.** The status of the indicated proteins in cells transfected with miR-30e or a control miRNA (miR-Con) was determined by Western Blot analyses at the indicated time points following exposure to γIR or etoposide. Representative blots from three to six independent experiments are shown. Densitometric and statistical analyses of the protein expression can be found in Suppl. Figures 4 and 5. Please note that the p21 blot shown in D was overexposed to better visualize the *per* se much lower p21 expression levels in irradiated p53-deficient cells. For a direct comparison of γIR-induced p21 protein levels in p53-deficient and p53-proficient HCT116 cells, please compare lanes 3 and 7, respectively, that are both derived from the same gel/blot and were thus scanned at the same intensities.

In order to search for miR-30e targets that could be held responsible for the observed effects, we first compared expression of apoptosis- and cell cycle-related proteins in irradiated HCT116 cells transfected with miR-30e or a control miRNA. Consistent with a recent report [30], we also found that miR-30e negatively regulates expression of the E2-conjugating enzyme Ubc9 (Figure 4A; Suppl. Figure 4). With regard to expression of other potential miR-30e targets such as procaspases 2, 8 and 9, as well as Bcl-2, Bax, and Bak, no or only marginal differences were observed between untreated and irradiated cells expressing miR-30e or the control miRNA (Figure 4A; Suppl. Figure 4). In contrast, regardless of whether or not HCT116 cells were exposed to γIR or etoposide, cells overexpressing miR-30e displayed a substantial downregulation of procaspase-3 compared to cells transfected with the control miRNA (Figures 4B, 4C; Suppl. Figure 5A left and middle panel). In addition, and most astonishingly, overexpression of miR-30e greatly increased DNA damage-induced protein levels of p21 (Figures 4B, 4C; Suppl. Figure 5B, left and middle panel). As DNA damage-induced p53 expression was not significantly affected by miR-30e (Figures 4B, 4C; Suppl. Figure 5C), and because miR-30e induced up- and downregulation of p21 and procaspase-3 expression, respectively, also in irradiated p53-deficient HCT116 cells (Figure 4D; Suppl. Figures 5A and 5B, right panels), our data suggest that these events occur in a p53-independent manner. In addition, and consistent with our finding that miR-30e accelerates senescence also in HCT116 cells exposed to lower γIR doses (Suppl. Figure 2C), miR-30e-induced modulation of p21 and procaspase-3 expression was also detected in HCT116 cells irradiated with only 2 Gy (Suppl. Figures 2D-F).

Overexpression of miR-30e in RKO cells had also no effect on γIR- or etoposide-induced p53 expression, but diminished procaspase-3 expression regardless of whether or not these cells were exposed to DNA-damaging agents (Figures 4E, 4F; Suppl. Figures 5A and 5C, left and middle panels). These findings are similar to those obtained in HCT116 cells and are thus consistent with the ability of miR-30e to inhibit DNA damage-induced caspase-3-like DEVDase activities in several cell lines (Figures 3C, 3D). In contrast to the observed miR-30e-mediated p21 increase in DNA-damaged HCT116 cells, however, miR-30e was unable to exert this effect in similarly treated RKO cells (Figures 4E, 4F; Suppl. Figure 5B, left and middle panel). Again, these data demonstrate differential target usages of miR-30e in HCT116 *vs.* RKO cells, and are thus in full agreement with the inability of miR-30e to rescue RKO cells from DNA damage-induced death and to induce senescence.

**Figure 5:**
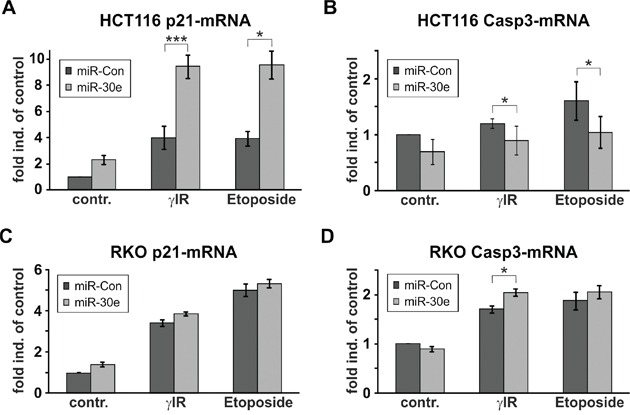
MiR-30e increases p21-mRNA expression only in HCT116 cells **A-D.** Real-time PCR for the determination of the expression levels of the p21-mRNA (A, C) and caspase-3-mRNA (B, D) in HCT116 wild-type (A, B) and RKO cells (C, D) that were transfected with miR-30e or a control miRNA (miR-Con). Total RNA was isolated 24h following exposure to γIR or etoposide and analyzed with transcript-specific probes from Applied Biosystems. Data shown represent the mean of three independent experiments +/− SD.

To examine possible effects of miR-30e on caspase-3- and p21-mRNA transcripts, we performed real-time PCR analyses with isolated RNA samples from both cell lines transfected with miR-30e or a control miRNA. Levels of both transcripts were hardly affected by miR-30e in RKO cells (Figures 5C, 5D). In contrast, whereas overexpression of miR-30e slightly lowered caspase-3 transcripts in DNA-damaged HCT116 cells (Figure 5B), it induced a massive upregulation of p21-mRNA particularly in HCT116 cells exposed to γIR or etoposide (Figure 5A).

**Figure 6:**
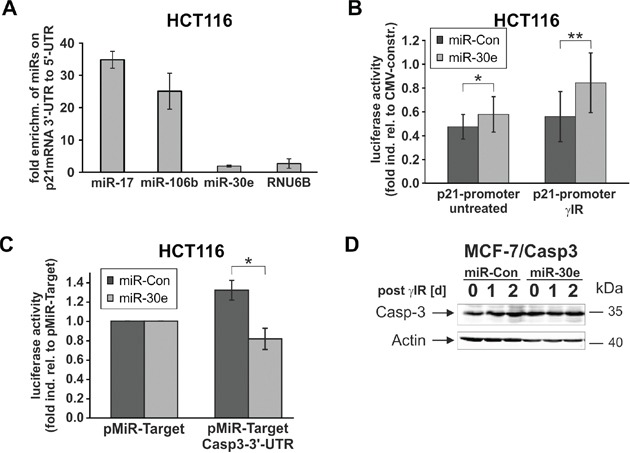
MiR-30e exerts translational and transcriptional effects on caspase-3 and p21 expression, respectively **A.** Real-time PCR analyses show that miR-30e does not bind to the p21-mRNA. Cellular extracts of untreated HCT116 wild-type cells were incubated for 2 h with BrU-labelled p21-mRNAs containing either the 3′-UTR or 5′-UTR in addition to the coding sequence. Following pulldowns of the p21 mRNAs with a BrdU antibody, the eluates were analyzed by real-time PCR for the presence of the indicated miRNAs. Data shown are the mean of two independent experiments +/− SD and represent the fold enrichment of miRNAs precipitated with 3′-UTR-p21-mRNA in relation to the amount pulled down with the 5′-UTR-p21-mRNA. **B, C.** Reporter gene assays show that miR-30e increases expression of a luciferase construct containing the p21 promoter (B), but represses luciferase expression in the presence of the 3′-UTR of the caspase-3-mRNA (C). The values are derived from three (C) and six to seven (B) independent experiments +/− SD. **D.** MiR-30e is unable to repress caspase-3 protein expression in MCF-7/Casp3 cells lacking the 3′-UTR. Western blot for the status of procaspase-3 was determined at the indicated times in irradiated MCF-7/Casp3 cells that were transfected with miR-30e or a control miRNA (miR-Con). One representative blot out of three is shown. Please note that the densitometric analysis of this blot is shown in Suppl. Figure 5D.

To more closely examine this intriguing result, we asked whether miR-30e mediates p21 upregulation by directly binding to the 3′-UTR of the p21-mRNA. Therefore, we incubated extracts of untreated HCT116 wild-type cells with bromouridine (BrU)-labeled p21-mRNAs containing either the 3′-UTR or the 5′-UTR. Subsequent real-time PCR analyses demonstrated that only miR-17 and miR-106b, two miRNAs known to inhibit p21-mRNA translation by binding to its 3′-UTR [10], were greatly enriched (∼ 35- and 25-fold, respectively) in eluates from 3′-UTR-p21-mRNA pulldowns when compared to eluates derived from precipitation of the 5′-UTR-p21-mRNA (Figure 6A). In contrast, neither miR-30e nor RNU6B, a small nuclear RNA involved in splicing of pre-mRNAs and thus unable to interact with mature mRNAs, were found enriched in 3′-UTR-p21-mRNA eluates. (Figure 6A). On the other hand, miR-30e increased expression of a luciferase construct containing the p21 promoter both in untreated and in irradiated HCT116 wild-type cells (Figure 6B). Together, these results strongly suggest that miR-30e upregulates p21 expression not at the translational level by direct binding to its 3′-UTR, but rather indirectly most likely via repression of factors regulating p21 transcription.

In contrast, reporter gene assays demonstrate that miR-30e downregulates procaspase-3 expression directly at the translational level. Cotransfections of miR-30e together with luciferase vectors that either contain or lack the 3′-UTR of the caspase-3-mRNA show that miR-30e inhibits luciferase expression only in the presence of the caspase-3 3′-UTR, but not in its absence (Fig. 6C). Together, with our finding that miR-30e is unable to suppress procaspase-3 expression in MCF-7/Casp3 cells (Figure 6D; Suppl. Figure 5D) which express caspase-3 only ectopically in the absence of the 3′-UTR [31], these results indicate that miR-30e regulates caspase-3 expression directly at the translational level. In summary, the identification of caspase-3 and p21 as direct and indirect miR-30e targets that are differentially regulated in HCT116 and RKO cells, explains our observation that only the former, but not the latter cells, can be rescued from DNA damage-induced cell death and driven into senescence.

## DISCUSSION

In order to identify senescence-associated miRNAs, we performed a microarray screen using p53-proficient and -deficient HCT116 colon cancer cells that upon irradiation are driven into senescence and apoptosis, respectively. Several miRNAs were found upregulated specifically in senescent wild-type cells including miR-30c, miR-30e, miR-34a, and miR-193b, whereas expression of miR-7 and miR-484 was diminished when compared to apoptotic p53-deficient cells. Curiously, expression of the other miR-30 family members remained unaffected by this treatment. This is probably due to the fact that miR-30c and miR30e are located together in a cluster within intron 5 of the NFYC gene on chromosome 1p34.2 and are thus commonly up- or downregulated together [32, 33], whereas miR-30a, -30b and -30d are transcribed from chromosomes 6q13 and 8q24.22, respectively. There is another gene copy of miR-30c clustered together with miR-30a on chromosome 6, but because miR-30a is not found upregulated in our experimental setting, it is unlikely that this gene copy is responsible for the observed induction of miR-30c expression.

So far, multiple cell models have been utilized to study γIR-induced miRNA expression changes, however, inconsistent results among these cell lines indicate that the regulation of some miRNAs after DNA damage might be cell type specific [34]. For example, a subset of miRNAs were responsive to γIR in A549 non-small cell lung cancer cells, but not in HCT116 cells [35, 36]. Also expression of miR-30c was found upregulated in irradiated human CD34+ hematopoietic progenitor cells, but inhibited in similar treated human fetal osteoblasts [37]. Even miRNA screens performed under almost identical conditions using identical cell lines may not always produce identical results. Whereas we succeeded in identifying miR-34a and miR-30e as crucial determinants in γIR-induced stress responses of HCT116 cells, another study identified only miR-34a, but none of the miR-30 family members among 36 miRNAs differentially expressed upon γIR in this cell line [35]. The exact reasons for this discrepancy are unknown, but probably manifold including different selection procedures, as Shin *et al*. [35], in contrast to our study, excluded miRNAs with expression levels changed less than 2-fold.

Several of the differentially expressed miRNAs identified here were previously shown to be involved in diverse stress responses including apoptosis and senescence, further validating the specificity of our screen [16, 29, 38–40]. For the following reasons we focused our attention mainly on the function of miR-30e. Firstly, together with miR-34a and miR-30c, miR-30e was specifically upregulated by γIR only in senescent cells such as wild-type HCT116 cells and HDF, but not in p53-deficient HCT116 cells or in RKO cells which both undergo apoptosis upon γIR, suggesting a senescence-associated expression pattern. Secondly, only miR-30e and miR-30c, but none of the other here identified miRNAs, were also found upregulated in another miRNA screen (data not shown) using MCF-7 breast carcinoma cells that undergo senescence following depletion of the centrosomal protein TACC3 [41]. Finally, miR-30e belongs to a miRNA family known to be involved in cellular senescence driven by the retinoblastoma protein [27], and whose members are postulated to mainly target mRNAs of proteins involved in apoptosis, senescence and cell cycle control [28].

Members of the miR-30 family have been regularly found downregulated in different types of cancer including breast cancer, ovarian cancer, lung cancer, hepatocellular carcinoma and multiple myeloma, suggesting a tumor suppressive role for this miRNA family [42–46]. Accordingly, whereas enforced expression of miR-30s in breast tumor-initiating cells (BT-IC) inhibited their self-renewal capacity, caused apoptosis and prevented tumorigenesis by targeting Ubc9 and integrin β3, miR-30 antagomirs efficiently counteracted these events [47, 48]. Also the recent identification of targets such as p53, perforin, Bim, K-Ras, Rab18 and Bcl9 which are suppressed by miR-30s directly *via* binding to their 3′-UTRs further links miR-30 family members to apoptosis and cell cycle control [46, 49–54]. In line with these findings, we also report here the identification of two additional and novel miR-30e targets, namely procaspase-3 and p21, that are both critical determinants in these processes. Whereas the caspase-3-mRNA represents clearly a direct miR-30e target that becomes translationally repressed following binding of miR-30e to its 3′-UTR, the observed upregulation of p21-mRNA and protein appears to be quite puzzling, as it is contrary to the common belief that miRNAs usually cause gene silencing by translational repression or degradation of their target mRNAs [55].

Although thus rather untypically, a few reports demonstrated an upregulation of proteins following overexpression of miRNAs, but these effects were shown or generally believed to be of an indirect nature [56–58]. However, upregulation of Bcl-2 and IL-10 for instance was achieved by direct binding of miR-21 and miR-466I to their respective 3′-UTR mRNAs [59, 60]. Whereas the mechanism for the miR-21-mediated Bcl-2 upregulation remains obscure, the finding that miR-466I competes with the RNA-binding protein (RBP) tristetraprolin for the same binding site in the AU-rich 3′-UTR of the IL-10-mRNA was suggested as plausible explanation for the observed upregulation of this cytokine [60]. This is because miRNAs mediate target mRNA degradation or translational repression only modestly [61], whereas RBPs such as tristetraprolin cause rapid mRNA destruction [62], resulting in an extended half-life of IL-10 mRNA and elevated IL-10 protein expression. As miRNAs are even able to upregulate protein expression in a cell cycle- and position (5′-UTR)-dependent manner, it is evident that miRNAs are able to exploit numerous mechanisms to increase protein expression [63, 64].

However, the here observed p21 upregulation is clearly not mediated by a direct binding of miR-30e to the p21-mRNA, as real-time PCR analyses of 3′-UTR-p21-mRNA immunoprecipitates confirmed only the presence of miRNAs known to silence p21 expression such as miR-17 and miR-106b [10], whereas miR-30e was conspicuously absent in these eluates. This is consistent with algorithms from miRanda and TargetScan data bases that revealed no obvious binding sites for miR-30 family members in the p21-mRNA. Thus, it appears that miR-30e upregulates p21 in an indirect manner perhaps by interfering with the expression of RBPs such as Musashi or the two heterogeneous nuclear ribonucleoproteins (hnRNP) K and D (AUF1) that are known to result in p21-mRNA degradation [10]. Because mRNAs of these RBPs do also not harbor miR-30e recognition sites, we favor another alternative in which miR-30e represses factors that directly or indirectly control p21 transcription e.g. p53, c-Myc, T-box factor-2, or c-Jun [10]. Our experiments exclude an involvement of p53 in this process, as miR-30e had no effect on p53 levels and induced upregulation of p21 even in p53-deficient HCT116 cells. Being particularly aware of the versatile mechanisms by which the remaining factors and probably also many other factors not listed above inhibit p21 transcription (e.g. direct repression at the p21 promoter; competing with other transcription factors for p21 promoter binding; direct inhibition of p21-inducing transcription factors), it will be quite challenging to identify the miR-30e-targeted p21-regulating factor(s). To succeed in this search it might perhaps help to consider genomic and proteomic differences between HCT116 and RKO cells, as miR-30e upregulates p21 only in the former but not in the latter cell line. In this matter it is interesting to note that knockout of the transcription factor ZBED6 (zinc finger, BED-containing-6), which modulates gene expression primarily by repressing transcription, resulted in opposing effects in these two cell lines. Whereas HCT116 cells displayed growth retardation upon ZBED6 knockout, RKO cells responded with increased growth rates [65]. Although an effect on p21 transcription was not evaluated, several ZBED6 target genes identified were linked to the Wnt/β-catenin and the phosphatidylinositol-3-kinase (PI3K)/Akt pathways, both of which are not only involved in colorectal cancer development, but also in the control of p21 expression [66, 67]. Interestingly, both cell lines harbor an activating mutation in the PIK3CA gene, encoding the p110a catalytic subunit of class I PI3Ks. In contrast, only HCT116 cells exhibit an additional mutation in the β-catenin gene resulting in an overall different proteomic response of both cell lines towards Wnt signaling [68]. As components of both the Wnt/β-catenin and the PI3K/Akt pathways are targeted by members of the miR-30 family [46, 54, 69], one might speculate that the sought-after miR-30e-targeted p21-regulating factor(s) might belong to these signaling cascades. Alternatively, by targeting K-Ras [52], as well as B-Raf [70], miR-30 family members also interfere with another tumorigenic pathway (Ras/B-Raf/MEK/ERK) that is implicated in the occurrence of colorectal cancer and that also regulates p21 expression by controlling multiple transcription factors including c-Myc, Mitf and Sp1, as well as members of the forkhead box O (FoxO), Ets, and CCAAT/enhancer-binding-protein (C/EBP) families [71]. Intriguingly, HCT116 cells harbor an activating mutation in the apical K-Ras^G13D^ gene, whereas RKO cells are characterized by a hyperactive B-Raf^V600E^ mutant. As both oncogenes differ greatly in their tumorigenic potential resulting in different gene expression profiles as well as in the induction of different senescence programs [72, 73], it is tempting to speculate that miR-30e targets a factor(s) involved in this pathway. Nevertheless, whatever the nature of this unknown factor(s) might be, targeting miR-30e may overcome therapy resistance of tumors that respond to DNA damage with upregulation of miR-30e.

## MATERIALS AND METHODS

### Cell lines, reagents and antibodies

HCT116 wild-type and p53-deficient colon carcinoma cells were maintained in McCoy's 5A medium (PromoCell, Heidelberg, Germany), whereas RKO colon carcinoma cells were cultured in high glucose DMEM (Gibco, ThermoFisher Scientific, Waltham, MA, USA). All cell lines were authenticated by DNA fingerprinting (DSMZ, Braunschweig, Germany) and are routinely tested for mycoplasma contamination. Human dermal fibroblasts (HDF) [74] were cultured in DMEM low glucose supplemented with 1% MEM nonessential amino acids (both Gibco) and 0.1 mM β-mercapto-ethanol (Carl Roth, Karlsruhe, Germany). Only passages below P20 were used. All media were supplemented with 10% heat-inactivated fetal bovine serum (Biowest SAS, Nuaille, France), 10 mM glutamine, 100 U/ml penicillin and 0.1 mg/ml streptomycin (Biochrom GmbH, Berlin, Germany). The fluorogenic caspase-3 substrate DEVD-AMC (N-acetyl-Asp-Glu-Val-Asp-aminomethylcoumarin) was from Biomol (Hamburg, Germany). The polyclonal caspase-3 antibody was from R&D Systems (Wiesbaden, Germany), and polyclonal caspase-9 and Ubc9 antibodies were from Cell Signaling Technology (Danvers, MA, USA). Caspase-2 and caspase-8 mAbs were from Enzo Life Sciences GmbH (Lörrach, Germany). From BD Biosciences (Heidelberg, Germany) we purchased the monoclonal p21 antibody, whereas the p53 monoclonal Ab-6 antibody was from Calbiochem (Bad Soden, Germany). The monoclonal Bcl-2 antibodies were from Santa Cruz (Heidelberg, Germany), whereas the monoclonal rabbit antibodies recognizing Bax and Bak were purchased from Upstate Biotechnology (Dublin, OH, USA). The monoclonal β-actin antibody as well as propidium iodide, etoposide, the protease inhibitors PMSF, aprotinin, leupeptin and pepstatin were from Sigma-Aldrich (Deisenhofen, Germany). Infrared fluorescence-labeled secondary antibodies were from Li-Cor Biosciences (Lincoln, Nebraska, USA).

### Treatment of cells and determination of cell death and senescence-associated β-galactosidase (SA-β-Gal) activity

If not otherwise stated, cells were exposed routinely to 20 Gy γIR (at 200 kV) using a Gulmay RS225 X-ray system from X-Strahl (Camberley, UK), or were treated with etoposide (50 μM) for the indicated times. Cell death was assessed cytometrically by the uptake of propidium iodide (2 μg/ml in phosphate-buffered saline) to determine the percentage of cells showing a loss of membrane integrity. Flow cytometric analyses were performed on a LSR-Fortessa (Becton Dickinson, Heidelberg, Germany) using the BD FACSDiva analysis software. For each determination, a minimum of 10 000 cells was analysed. For the detection of SA-β-Gal activity, cells were fixed in 2% formaldehyde and 0.2% glutaraldehyde for 5 minutes at RT, washed in PBS, and incubated at 37°C in the absence of CO_2_ in staining solution (150 mM NaCl, 2 mM MgCl_2_, 5 mM K_3_Fe(CN)_6_, 5 mM K_2_Fe(CN)_6_, 40 mM citric acid, and 12 mM sodium phosphate [pH 6.0]) containing 1 mg/mL 5-bromo-4-chloro-3-indolyl-D-galactoside. Digital pictures were taken on an Axio Observer A1 microscope with a 10x objective using AxioVision Software (Carl Zeiss MicroImaging GmbH, Göttingen, Germany).

### Preparation of cell extracts, western blotting and fluorometric determination of caspase activity

Total cell extracts were prepared in lysis buffer containing 1% NP-40, 50 mM Tris-HCl (pH 7.4), 150 mM NaCl, 1 mM DTT, and protease and phosphatase inhibitors. Protein concentrations were determined with the BioRad protein assay. Proteins were separated on SDS-polyacrylamide gels and electroblotted to polyvinylidene difluoride membranes (Millipore, Schwalbach, Germany). Following primary antibody incubation, proteins were visualized by infrared fluorescence-labelled secondary antibodies using the Li-Cor Odyssey imaging system (Lincoln, Nebraska, USA). Caspase-3-like DEVDase activities were assessed as described and presented as arbitrary units (AU) [9].

### Luciferase reporter assay

To determine the influence of miR-30e on caspase-3 expression, cells were transfected using DharmaFECT Duo (Dharmacon, Lafayette, CO, USA) with the pMiR-Target vector containing the firefly luciferase cDNA with or without the 3′-UTR of the caspase-3-mRNA (Origene Technologies, Rockville, MD, USA) together with miR-30e or a non-targeting control miR. To determine the influence of miR-30e on p21 expression, cells were transfected with a firefly luciferase reporter plasmid under the control of either the CMV or the p21 promoter together with miR-30e or the control miR. In both setups, a renilla luciferase construct (pRL-TK) was co-transfected for normalization purposes. Cells were either left untreated or irradiated 24 h post transfection, and firefly and renilla luciferase activities were determined another 24 h later with the dual-luciferase reporter assay (Promega, Fitchburg, WI, USA) and a Centro LB960 luminometer (Berthold Technologie, Bad Wildbad, Germany).

### p21-mRNA immunoprecipitation

To analyze binding of miRNAs to p21-mRNA, bromouridine (BrU)-labeled p21-mRNAs containing the 5′- or 3′-UTR in addition to the coding sequence were generated using the mMESSAGE mMACHINE® T7-transcription kit according to the manufacturer's protocol (ThermoFisher Scientific). Following a 2 hour incubation of the BrU-labeled p21-mRNAs with HCT116 wild-type cell extracts, immunoprecipitations were carried out with the RiboTrap kit according to the manufacturer's protocol (MBL, Woburn, MA, USA). Total RNA was isolated from the eluates with the miRNeasy kit (Qiagen, Hilden, Germany). Relative microRNA expression levels of the inputs and eluates were quantified by real-time PCR.

### Real-time PCR

To determine the expression levels of miRNAs, the miRNeasy kit from Qiagen was used to isolate the cellular small RNA fraction. MiRNA-specific cDNAs were generated using the microRNA reverse transcription kit with specific hairpin primers supplied with the microRNA TaqMan assay (Life Technologies, Karlsbad, CA, USA). MicroRNA expression levels were quantified by real-time PCR using specific TaqMan probes (Life Technologies). The small RNAs RNU6B and RNU43 were used for normalization. To determine the influence of miR-30e on the expression of the p21-mRNA and caspase-3-mRNA, cells were transfected with miR-30e or a control miR and exposed to either γIR or etoposide. Total RNA was isolated with the RNeasy kit (Qiagen) and reverse transcribed with the High Capacity cDNA Kit (Life Technologies). Taqman gene expression probes for human p21, caspase-3, GAPDH and β-actin mRNAs (Life Technologies) were employed to analyse their relative expression levels using the 7300 Real-Time PCR system (Life Technologies). The β-actin- or GAPDH-mRNA served as an endogenous normalization control for every sample. Fold induction of the analyzed RNAs was calculated via the 2^(Δ(ΔCt))-method normalizing all samples to the level of the analyzed RNA in untreated miR-control-transfected cells.

### MicroRNA expression profiling

Three independent biological replicates of microRNAs isolated from untreated wild-type and p53-deficient HCT116 cells and from cells 16 and 72 hours post γIR (20 Gy) were analyzed by the miRNA expression profiling services from Dharmacon (microarray analysis with miRNAs listed in miRBase 11.0). Beforehand, the quality of the purifications was validated by us via real-time PCR for miR-34a upregulation and by Dharmacon via determination of the RNA integrity number (RIN).

### Transfection of miRNAs

MiRidian microRNA mimics and the non-targeting mimic control miRNA were purchased from Dharmacon RNA technologies. Transfections were carried out according to the manufacturer's instructions. Twenty-four hours after transfection with DharmaFECT1, cells were divided equally to receive either no treatment or exposure to γIR or etoposide. Cells were harvested at the indicated time points and analysed as indicated.

### Statistical analyses

When applicable paired student's t-test was performed for significance analyses. * p<0.05; ** p<0.01; *** p<0.001.

## SUPPLEMENTARY FIGURES


