# Supplementary material for: miR-30e controls DNA damage-induced stress responses by modulating expression of the CDK inhibitor p21WAF1/CIP1 and caspase-3
Source: Oncotarget. 2016 Feb 17;7(13):15915–29. doi: 10.18632/oncotarget.7432 (PMC4941286; doi:10.18632/oncotarget.7432)
Supplement: Supplementary file 1 [file oncotarget-07-15915-s001.pdf]

SUPPLEMENTARY FIGURES

A

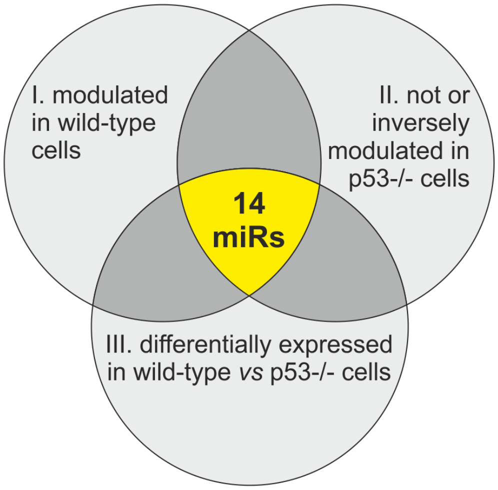

B

	Microarray			Real-Time PCR			
	HCT116 wild-type			HCT116 wild-type	HCT116 wild-type *vs.* p53-/-		
microRNA	fold ind.	p value	sign.	fold ind.	fold differ.	p value	sign.
hsa-miR-193b	1.25	0.0015	**	1.80	1.47	0.0362	*
hsa-miR-30c	1.18	0.0121	*	2.13	2.20	0.0168	*
hsa-miR-30e	1.28	0.0139	*	2.13	1.79	0.0106	*
hsa-miR-34a	1.54	0.0001	***	2.76	3.54	<0.0001	***
hsa-miR-484	0.72	0.0088	**	0.59	0.51	0.0036	***
hsa-miR-7	0.59	0.0001	***	1.04	0.65	0.0086	**

Supplementary Figure 1: Schematic illustration of the strategy employed to identify p53-dependent, senescence-specific microRNAs by microarray analyses. A. Firstly, only microRNAs displaying a significant up- or downregulation in HCT116 wild-type cells three days after γIR compared to untreated cells were evaluated (I). Secondly, from the miRs identified in I, only expression of those was considered to be p53-dependent which were not or inversely modulated in HCT116 p53-deficient cells during the same time frame (II). Finally, only miRs were considered to be expressed in a p53-dependent and senescence-specific maner if their overall expression on day 3 post γIR was significantly different between wild-type and p53-deficient HCT116 cells (III). **B.** The 14 microRNAs identified in this manner were further verified by real-time PCR, resulting in the validation of six p53-dependent, senescence-specific microRNAs. The values shown are the mean of at least three independent experiments. For a graphical data presentation, please see Fig. 1A and 1B. For statistical analysis, a paired students t-test was performed if applicable.

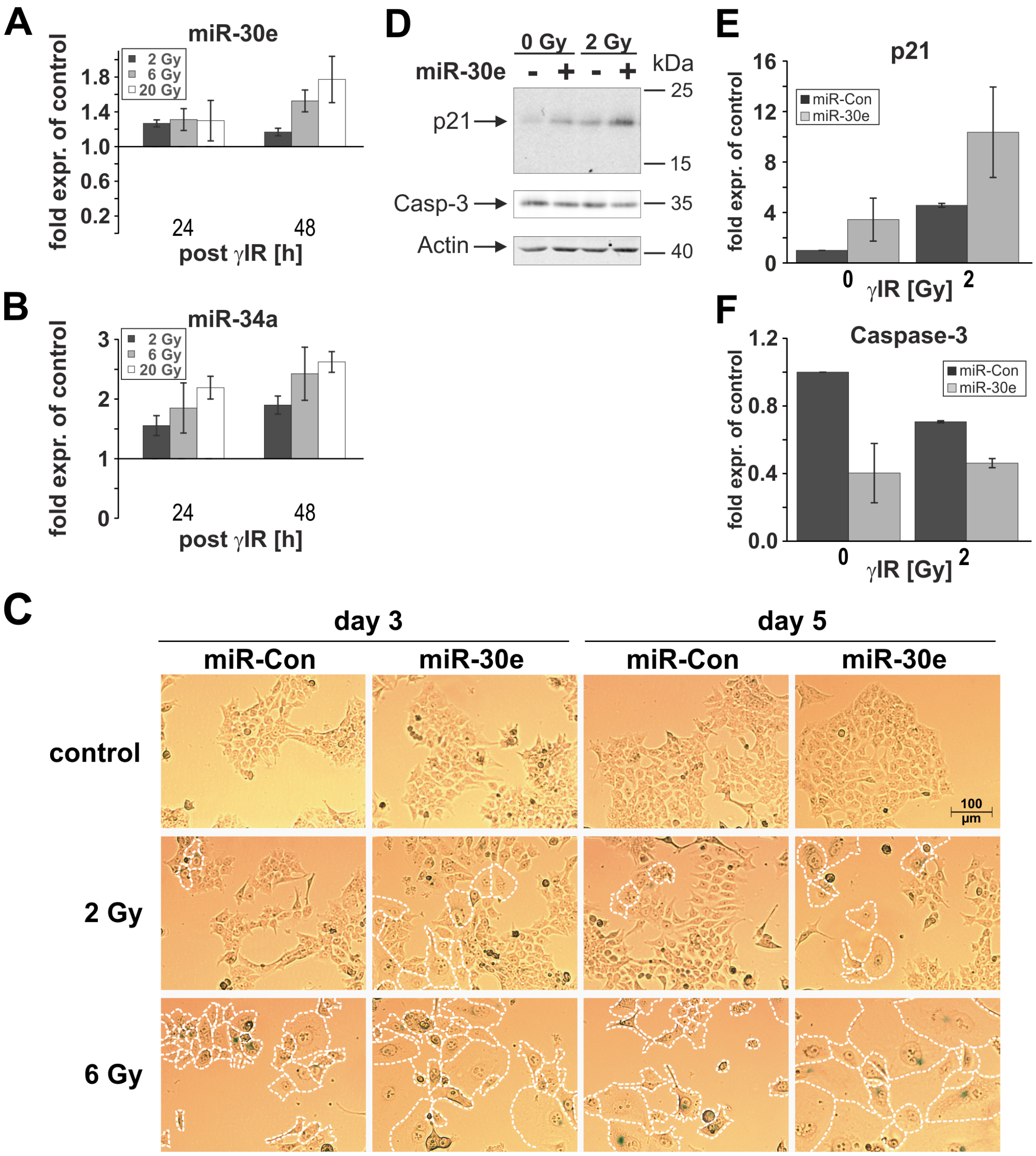

Supplementary Figure 2: Effects of miR-30e in concert with low-dose irradiation. A, B. Determination of miR-30e and miR-34a expression by real-time PCR following exposure of HCT116 wild-type cells to different γIR doses. Values shown are the mean of at least three independent experiments +/- S.D. **C.** Cells were transfected with the indicated miRNAs and were either left untreated (control) or exposed to different doses of γIR and analyzed for SA-β-Gal staining at the indicated times. To better illustrate size increases, senescent SA-β-Gal-stained cells were encircled with dotted lines. Representative pictures out of two independent experiments are shown. All pictures were taken at a 10x magnification (100 µm scale bar included). **D-F.** MiR-30e induces up- and downregulation of p21 and procaspase-3 expression following low dose (2 Gy) irradiation of HCT116 wild-type cells. The status of the indicated proteins in untreated or irradiated (2 Gy) cells transfected with miR-30e or a control miRNA (miR-Con) was determined by Western Blot analyses. Representative blots (D) and densitometric analysis (E-F) from two independent experiments are shown.

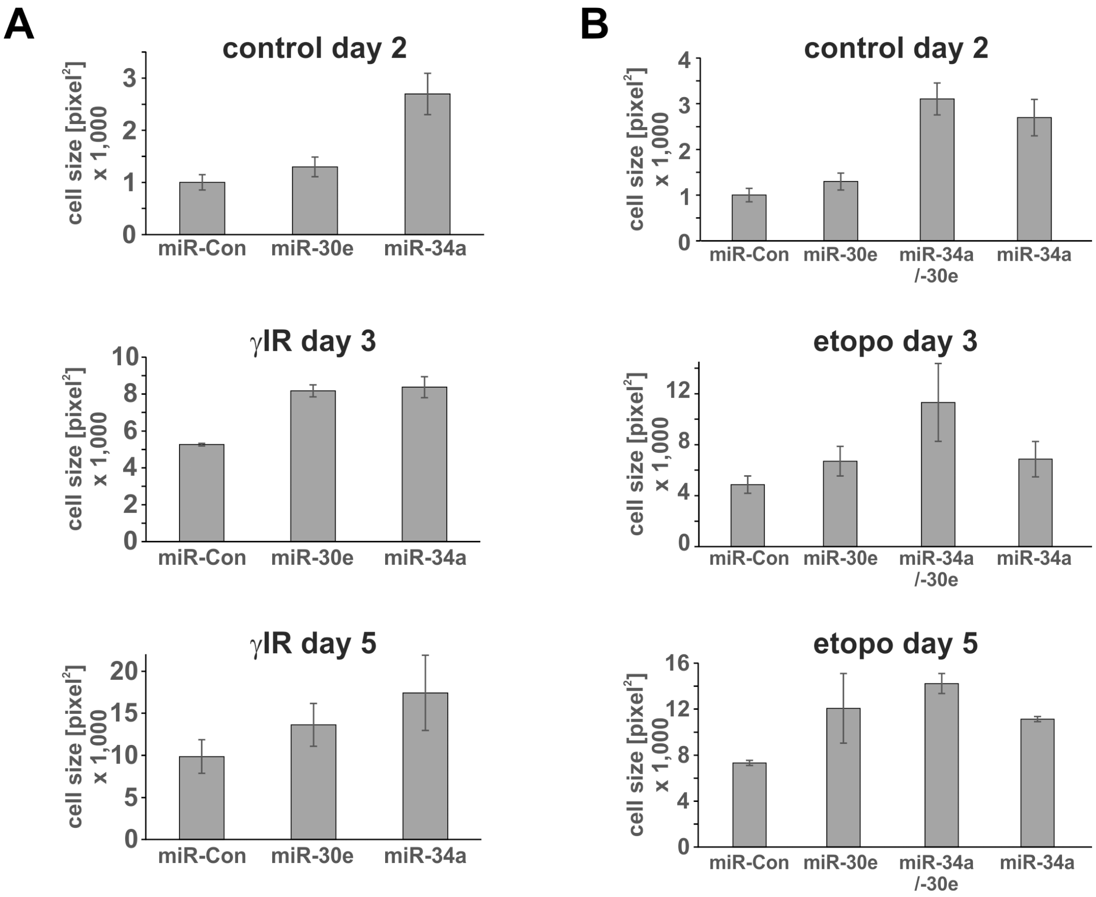

Supplementary Figure 3: Size analyses of senescent cells shown in Figure 2. A, B. Statistical determination of the cell size of γ-irradiated. (A) or etoposide-treated (B) HCT116 cells which were transfected with the indicated microRNAs before treatment. Values shown are the mean of two to three independent experiments +/- S.D. For every condition and experiment, the size of at least 200 cells was determined using the ImageJ software. Since all pictures were taken at the same magnification and resolution, cellular sizes were measured as $pixel^2$.

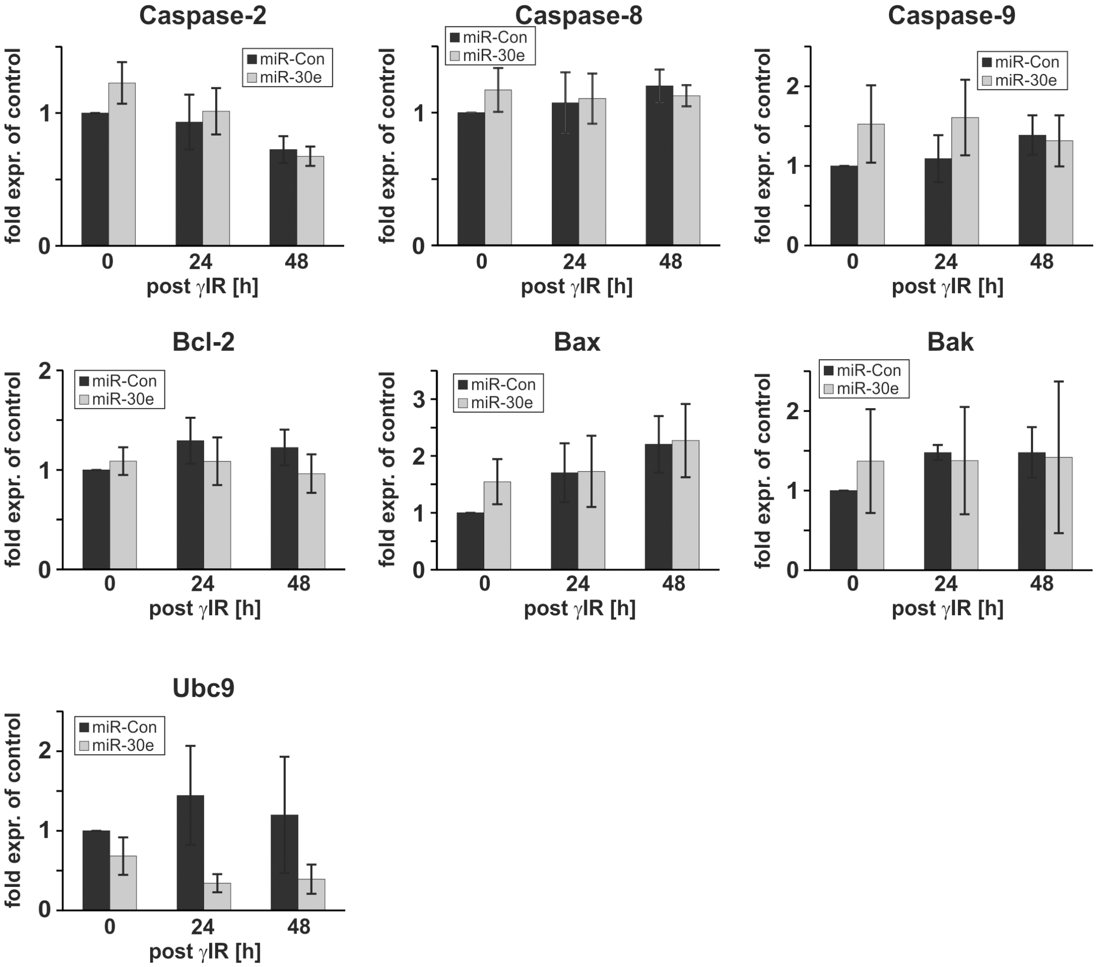

Supplementary Figure 4: Densitometric analysis of the protein expression shown in the western blots in Fig. 4A. HCT116 wild-type cells were transfected with a non-targeting control microRNA or miR-30e before they were exposed to 20 Gy γIR. At the indicated time points the cells were harvested and their cellular extracts were analyzed for expression of the indicated proteins. Densitometric analyses were performed using the Odyssey V3.0 or the ImageJ analysis software. Values shown are the mean of three to six independent experiments +/- S.D.

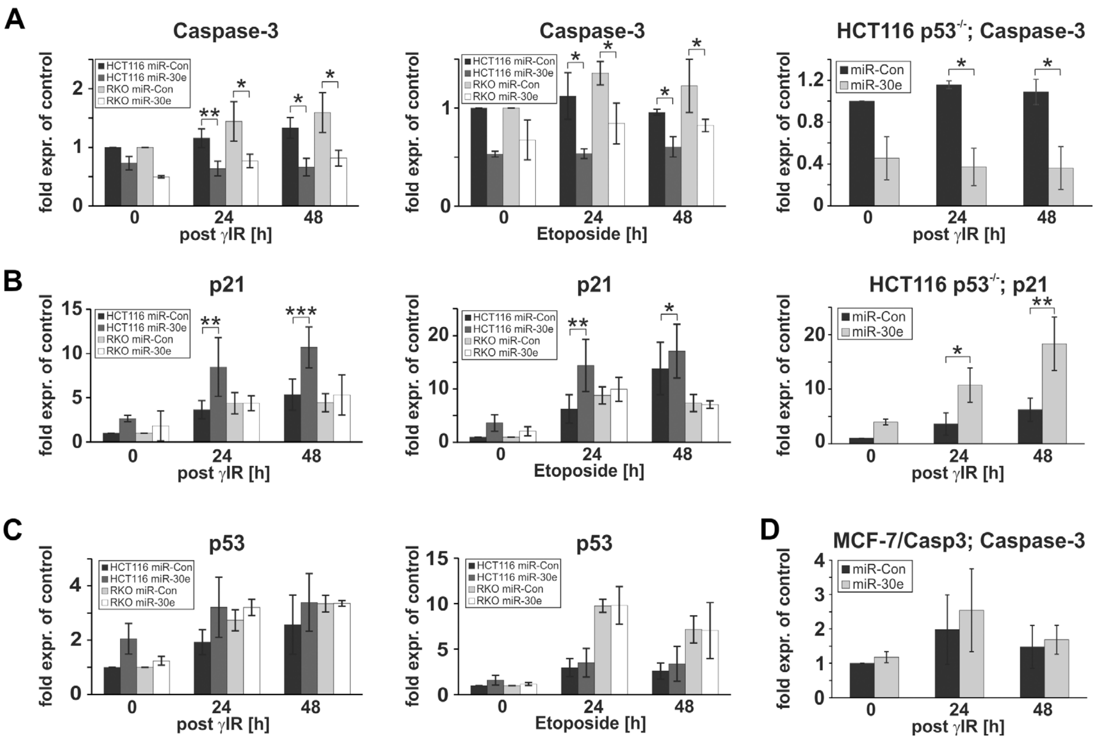

Supplementary Figure 5: Densitometric analysis of the protein expression shown in the western blots in Fig. 4B-F. A-D. HCT116 wild-type and RKO cells (A-B, left and middle panel; C), as well as HCT116 p53$^{-/-}$ (A-B, right panel) and MCF-7/Casp3 cells (D) were transfected with a non-targeting control microRNA or miR-30e before they were exposed to 20 Gy γIR. At the indicated time points after treatment the cells were harvested and their cellular extracts were analyzed by western blotting for expression of caspase-3 (A, D), p21 (B) and p53 (C). The densitometric analysis was performed using the Odyssey V3.0 or the ImageJ analysis software. Values shown are the mean of three to six independent experiments +/- S.D.
